# Wide-Awake Olecranon Tension Band Wiring with Ipsilateral Zone 6 Extensor Tendon Repair: A Case Report

**DOI:** 10.5704/MOJ.2203.023

**Published:** 2022-03

**Authors:** IJ Magtoto

**Affiliations:** Department of Orthopaedics, The Medical City Clark, Mabalacat, Philippines

**Keywords:** olecranon fracture, tension band wiring, WALANT

## Abstract

Wide-awake local anaesthesia, no tourniquet (WALANT) continues to gain popularity and has been adapted for various fracture fixations proximal to the hand. A case of olecranon tension band wiring with concomitant extensor tendon repair is presented. The procedure was done with the patient awake in a lateral decubitus position 30min after the injection of WALANT solution at the intended surgical sites. Pain and discomfort were felt by the patient towards the end of the procedure mainly on his volar forearm where the bolster was placed. Olecranon fixation under WALANT is a viable alternative for patients who prefer to be awake or those with contraindications to general or regional anaesthesia. Alternative patient positioning may be considered, and perioperative pain control should not be overlooked.

## Introduction

Wide awake local anaesthesia, no tourniquet (WALANT) continues to gain popularity as a viable and cost-effective alternative for various hand surgical procedures^1^. There has also been increased interest in its application to areas beyond the hand to include plate fixation of the olecranon^2^. Tension band wiring of the olecranon under WALANT has yet to be reported in literature and presents with a few additional considerations as compared to plating.

A case of tension band wiring of the olecranon with a concomitant tendon repair of an ipsilateral zone 6 extensor injury of the middle finger is presented.

## Case Report

A 31-year-old right-handed male sustained an open, transverse fracture of his left olecranon with a complete transection of the ipsilateral extensor digitorum communis (EDC) of the middle finger at zone 6 from a vehicular accident. He was initially seen at the emergency room where appropriate IV antibiotics, tetanus prophylaxis and local wound debridement was done. He was advised for admission and surgical fixation, but he opted to be discharged to avoid admission due to worries of the possibility of contracting nosocomial COVID-19 infection amidst the ongoing pandemic. He was seen by the operating surgeon and the option for fixation and tendon repair under WALANT as an outpatient procedure was offered.

The solution for WALANT was prepared by mixing 25ml of 2% lidocaine, 75ml of plain normal saline solution, 0.5ml of epinephrine and 5ml of 8.4% sodium bicarbonate to produce approximately 105ml of buffered 0.5% lidocaine with 1:200,000 epinephrine solution. A total of 70ml of solution was injected at the operative site. The soft tissue around the surgical site was noted to be swollen, making palpation of the proximal ulna and deep infiltration of the anaesthesia towards the periosteum difficult. Additional anaesthesia was also placed distally to account for the intended length of the intramedullary placement of the k-wires. A 20ml of solution was placed on the dorsum of the hand for the extensor tendon repair. The remaining 15ml of the solution was placed on standby for intra-operative use. A waiting period of 30min was ensured prior to skin incision. A single IV dose of 2g of Cefazolin was also given prior to skin incision.

**Fig. 1:**
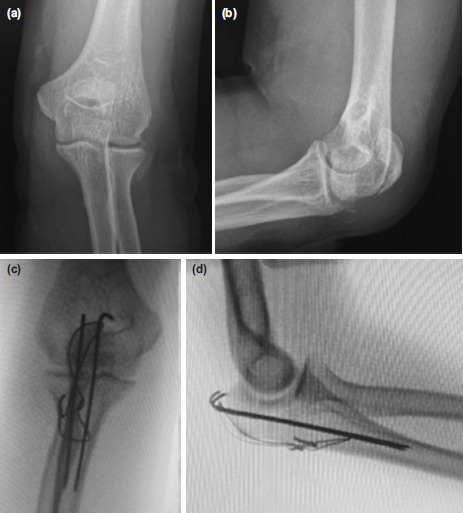
Case radiographs. (a) Pre-operative elbow AP and (b) Lateral views. (c) Final elbow AP and (d) Lateral views.

**Fig. 2:**
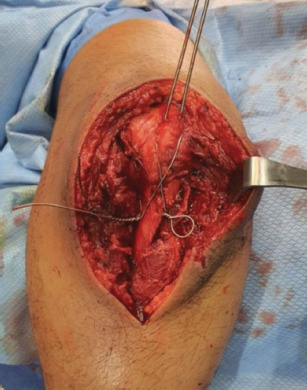
Intra-operative image prior to bending and cutting of wires showing fixation with manageable bleeding.

A posterior approach was done for access to the olecranon with the patient on right lateral decubitus position with his left elbow flexed on a bolster. The exposure was uneventful until dissection of the medial tissues elicited pain, prompting to add additional 10ml of solution around the medial periosteum. Tension band wiring with placement of intramedullary k-wires was uneventful with no reported pain (Fig. 1). Intra-operative active elbow extension was done to confirm the stability of fixation. Bleeding was manageable without the need for a tourniquet or electrocautery (Fig. 2).

**Fig. 3:**
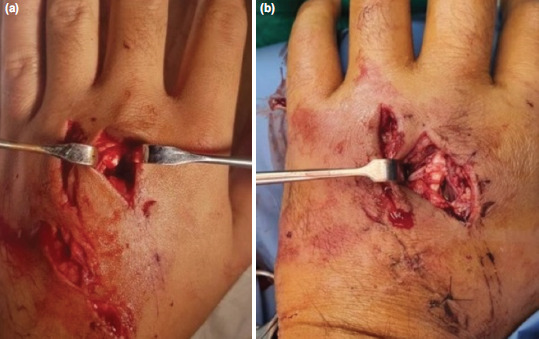
Extensor tendon injury. (a) Shows transected EDC of the left middle finger. (b) Shows the repair prior to the placement of the epitendinous suture.

During skin closure of the elbow, the patient started to feel vague pain at the proximal forearm near the area of the bolster with increasing severity. Intravenous Paracetamol 1g was given which provided minimal relief. An additional IV dose of Parecoxib 40mg was given which then provided relief. His middle finger EDC was repaired with a cruciate four-strand repair using 4-0 nylon and augmented with a running interlocking horizontal mattress suture using 6-0 nylon (Fig. 3). Intra-operative testing showed full range of motion with no repair gapping. Copious irrigation and closure of the remaining wounds were done. He was then discharged with oral pain medications. Follow-up at three months showed good hand and elbow function with no signs of infection.

## Discussion

Plate fixation of the olecranon under WALANT was initially described by Ahmad *et al*^2^. The technique for tension band wiring is similar with a few caveats. The more common pin configuration for the k-wires would be oblique starting from the proximal dorsal aspect of the olecranon with penetration towards the anterior cortex. This would necessitate additional infiltration of local anaesthetic on the anterior periosteum which would be difficult as it is surrounded by the flexor-pronator mass. Therefore, an intramedullary placement of the k-wires seems to be more practical. It must be noted that the intramedullary canal also has nociceptors, which means that infiltration of the anticipated area of the k-wire with local anaesthesia should also be done via the surrounding periosteum. Infiltration of the anterior periosteum may be possible and more accurate if the surgeon is trained to do ultrasound-guided injections. An ultrasound guided periosteal sleeve block may be considered.

An alternative positioning of the patient in supine may be considered and may be more tolerable for olecranon fixation under WALANT. The pain experienced by the patient on his antecubital area and proximal forearm may be related to his positioning with pressure being placed by the bolster, rather than inadequacy of the local anaesthetic. One theory may also be that localised hyperalgesia of the surrounding tissues near the operative site, in this case the volar tissues, was caused by peripheral sensitisation^3^. Pre-emptive analgesia has been suggested to address this^4^. As we continue to do more extensive bony procedures under WALANT, it would be prudent to have a protocol for pain management ready, similar to the one presented by Kelley *et al*^5^. Their approach classifies patients according to the risk of developing pain depending on the type of surgery and other patient related factors and have corresponding recommendations for anaesthesia and medications. Additional medications like pre-operative Pregabalin or Gabapentin may be considered to improve pain control.

Olecranon tension band wiring under WALANT is a viable alternative to general or regional anaesthesia, provided that adequate pain control is achieved. Other concomitant injuries may also be done if the duration of the procedure and maximal dose of lidocaine permits. Proper patient selection and thorough discussion of what to expect during the surgery is important. Pre-emptive analgesia and perioperative IV pain medications may play a role to improve pain control and an alternative patient positioning in supine may also be considered.
